# A Review of the Application of Compliance Phenomenon in Particle Separation Within Microfluidic Systems

**DOI:** 10.3390/mi16101115

**Published:** 2025-09-29

**Authors:** Wei Wang, Jin Yan, Junsheng Wang, Yuezhu Wang, Ge Chen, Zihao Weng, Hongchen Pang, Xianzhang Wang, Dapeng Zhang

**Affiliations:** 1 College of Naval Architecture and Shipping, Guangdong Ocean University, Zhanjiang 524088, China; wangwei@gdou.edu.cn (W.W.);; 2 Guangdong Provincial Key Laboratory of Intelligent Equipment for South China Sea Marine Ranching, Guangdong Ocean University, Zhanjiang 524088, China; 3 Liaoning Key Laboratory of Marine Sensing and Intelligent Detection, Dalian Maritime University, Dalian 116026, China

**Keywords:** compliance phenomenon, microfluidic system, elastic materials, particle separation, wettability-engineered

## Abstract

Microfluidic chips made of polydimethylsiloxane (PDMS) have shown significant application potential in aquatic environments with high microbial density, such as “marine ranches”, due to their high-throughput, high-efficiency and high-precision detection capabilities. This technology can rapidly identify pathogenic microorganisms or harmful particles in aquaculture systems, thereby providing urgently needed innovative methods for implementing preventive measures and enhancing aquaculture productivity. By regulating the micro-nano scale channel structure, microfluidic technology can precisely control fluid flow patterns, offering new insights and effective solutions for microbiological research and the separation and analysis of particulate matter. This paper first provides a concise overview of the application of microfluidic chip technology in the analysis of marine microorganisms. Subsequently, it focuses on the “compliance” phenomenon in PDMS-based microfluidic systems, systematically reviewing the potential mechanisms, latest progress and impacts of compliance behavior in mechanically elastic materials such as PDMS. Additionally, this article also investigates the role of “compliance” in key processes of microfluidic technology application, including the capture, separation, enrichment and detection of microorganisms and particles. Moreover, the relationship between surface wettability engineering and compliance phenomena is also explored. We believe that this review will contribute to enhancing the understanding and control of the mechanical behavior of microfluids and the particles they carry within microfluidic systems, providing valuable theoretical insights and practical guidance for researchers in this field.

## 1. Introduction

As humans gradually develop and utilize marine resources and move towards the “deep blue”, the impact of development activities on the marine environment is inevitable. Such impacts often feedback to human society through various natural processes, forming a two-way interaction mechanism. Many scholars have studied the influence of human activities on a macro scale, such as the changes in sea-air interaction caused by human social production (for example, the variation in the El Niño-Southern Oscillation cycle due to greenhouse gas emissions [1]; as well as the impact of engineering construction on the regional marine physical and chemical environment on a meso scale (such as the local tidal current changes and ecological niche shifts caused by semi-submersible drilling platforms, reclamation wharfs, berth projects, and carbon and nitrogen oxide emissions from ships [2,3,4]. However, compared with the more obvious environmental changes mentioned above, there are also a wide range of microscopic particles in the ocean that are difficult to detect with the naked eye, including non-living microplastics [5] and living microorganisms such as microalgae, parasites, bacteria and viruses. These microscopic particles, through cumulative effects at the micro level, bring about qualitative changes from quantitative ones, profoundly influencing human production and lifestyle.

With the continuous development of Lab-on-Chip (LOC) technology, it has attracted extensive attention in the detection and analysis of microscopic particles that are difficult to observe with the naked eye. The emergence of microfluidic detection technology enables marine sensing systems to obtain data more accurately and intelligently, thereby achieving real-time processing and analysis of multiple parameters in the marine environment. Compared with traditional large-scale experimental equipment, the sensing systems constructed with microfluidic technology as the core not only have smaller volume and lower cost, but also consume less power, significantly improving the economy, in situ capability and real-time response ability of marine sensing systems [6]. To date, microfluidic technology has developed into an interdisciplinary research field covering chemistry, biology, fluid mechanics, medicine, materials science, electronics and mechanics. According to the different liquid manipulation methods, microfluidic technology can be classified into continuous flow microfluidic chips, droplet-based digital microfluidics, optical microfluidics, acoustic microfluidics, electrokinetic microfluidics and magnetically driven microfluidics, among others [7].

In the application of microfluidic technology to the study of the impact and detection of non-living marine micro and nano plastic particles, many experts and scholars have focused on the field of “the impact of marine micro and nano plastic particles on humans and ecosystems”. Due to the wide distribution of micro and nano plastics in the marine environment, their sources include but are not limited to activities such as ship transportation, marine industries, and coastal tourism (Cole et al., 2011) [8]. The plastics produced by these sources eventually enter the ocean, posing a threat to the survival of marine organisms. Microplastics not only have the characteristic of being difficult to degrade, but also can absorb pollutants, which may disrupt the structure and function of marine ecosystems (Sharma and Chatterjee, 2017 [9]; Kazour et al., 2019 [10]). For instance, microplastic particles are easily ingested by marine organisms and accumulate in tissues, the circulatory system, and the brain, causing chronic biological effects, including chemical impacts (such as inflammation, liver stress, growth inhibition) and physical damage (such as hindering movement due to polymer attachment to the body surface, blocking the digestive tract, etc.). At the same time, the use of microfluidic technology to separate and characterize micro and nano plastic particles to achieve continuous, controllable and selective operation has become an important direction in the field of microfluidic technology research. Among them, dielectrophoresis technology, due to its advantages such as no labeling, low sample consumption, and simple operation, has become one of the most widely used technologies in microfluidic platforms. For example, Zhang et al. (2018) [11] achieved the controlled separation of particles in a hybrid dielectrophoresis-inertial microfluidic chip by adjusting the applied voltage; Sarno et al. (2021) [12] demonstrated the application potential of dielectrophoresis technology in the efficient sorting of micro and nano plastic particles of different sizes and types on a microfluidic chip; Ma et al. (2021) [13] developed a centrifugal microfluidic chip with an integrated rectifier structure, achieving efficient separation of microplastic particles, providing a new technical path for the microfluidic processing of such particles.

Compared with the research on non-biological microplastic particles, the impact of marine microorganisms with life activity and dynamic changes on the marine environment and human society deserves more attention. These seemingly “insignificant” microorganisms play a key role in marine carbon cycling, nutrient cycling, and the response of ecosystems to climate change. They drive global biogeochemical cycles in a group form and serve as indicators reflecting the health of natural ecosystems (such as coral reef ecosystems) and artificial ecosystems (such as marine ranching areas). However, traditional analysis methods often fail to meet the requirements for capturing the in situ dynamic behavior of microorganisms in terms of temporal and spatial resolution, while also facing technical challenges such as complex sampling, low biomass, and variable environmental conditions [14,15,16]. Microfluidic technology, with its precise control of microenvironments, high-throughput single-cell analysis capabilities, and small-scale systems that can be deployed on-site, has become a revolutionary tool for studying marine microorganisms. This technology achieves efficient observation of the active and passive behaviors of marine microorganisms by manipulating fluids at the micrometer scale and building small-scale, automated, and high-throughput system platforms, significantly enhancing human understanding of these “microscopic life forms” [7,17,18].

**Figure 1:**
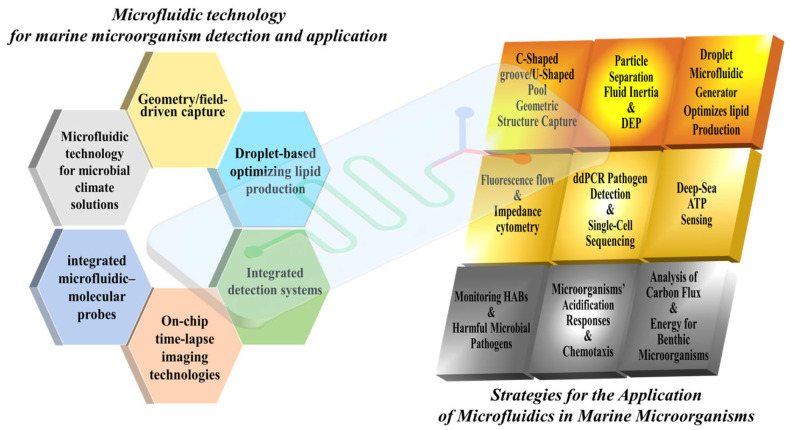
Technologies & application in microfluidic of marine microorganisms.

As shown in Figure 1, microfluidic technology has brought about a revolutionary change in the rapid detection and analysis of marine microorganisms by achieving miniaturization and high-throughput processing and detection capabilities. Its key technologies include:

(1) Geometry/field-driven capture techniques (such as C-shaped channel structures and dielectrophoresis);
(2) Droplet-based culture methods for optimizing lipid production;
(3) Integrated detection systems, covering fluorescence cell counting (based on chlorophyll autofluorescence), impedance cell counting (enabling label-free activity detection), and digital PCR and single-cell sequencing technologies (capable of detecting pathogens at a level of 10 copies per microliter and mapping host–virus interaction networks);
(4) On-chip time-lapse imaging technologies, by combining microfluidic chip technology with time-lapse imaging technology, high-resolution analysis of microbial behavior is achieved. This method enables real-time monitoring of microbial responses to various environmental gradients (such as pH, temperature and chemical signals) on a microfluidic platform;
(5) Integrated microfluidic probes can achieve rapid and highly sensitive detection in a variety of application scenarios. Take monitoring harmful algal behaviors such as HABs as an example (integrated microfluidic-molecular probes provide rapid and sensitive detection), ballast water compliance (with an accuracy rate exceeding 92%), and deep-sea ATP/gene detection;
(6) Microfluidic technology applied to microbial solutions for climate, such as microbial carbon and energy turnover functions (microfluidic technology clarifies the role of microorganisms in climate solutions).

**Figure 2:**
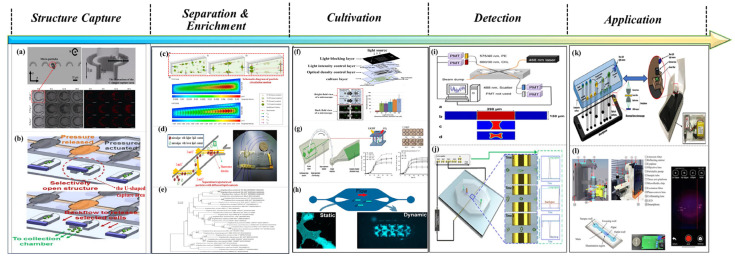
The main steps of marine microorganism detection based on microfluidics. (**a**) C-shaped Design for Enhanced Micro-particle Capture: Design and Experimental Characterization. Reproduced with permission from ref. [20]. Copyright (2025) MDPI AG. Reproduced with permission from ref. [19] Copyright (2013) Springer Nature. (**b**) Schematic diagram of U-shaped design enhancing micro-particle capture structure. Reproduced with permission from ref. [23] Copyright (2001) Royal Society of Chemistry. (**c**) CFD diagram of inertial microfluidics for microalgae separation. Reproduced with permission from ref. [31] Copyright (2018) Bioresource Technology. (**d**) Dielectrophoresis microfluidic chips for sorting and detecting lipid-variable microalgae. Reproduced with permission from ref. [22] Copyright (2014) AIP Publishing. (**e**) Microfluidic coating plate method for the isolation of different bacterial species. Reproduced with permission from ref. [30] Copyright (2018) Int. J. Syst. Evol. Microbiol. (**f**) High-throughput microfluidic light-controlled platform for biofuel microalgae analysis. Reproduced with permission from ref. [21] Copyright (2010) Royal Society of Chemistry. (**g**) Microdroplet photobioreactor for photoautotrophic microalgae culture. Reproduced with permission from ref. [24] Copyright (2016) Royal Society of Chemistry. (**h**) Microfluidic platform for biofilm formation and monitoring under microvortex flow. Reproduced with permission from ref. [25] Copyright (2024) American Chemical Society. (**i**) Design of Optical Analytical Microfluidic Cytometer. Reproduced with permission from ref. [27] Copyright (2011) Elsevier B.V. (**j**) Rapid and Precise Analysis of Marine Microalgae Using Microfluidic Impedance Cytometry. Reproduced with permission from ref. [26] Copyright (2024) Royal Society of Chemistry. (**k**) A 3D-printed smartphone platform with opt electrowetting microfluidics for automated on-site monitoring of viable algae in water. Reproduced with permission from ref. [28] Copyright (2019) Elsevier B.V. (**l**) A microfluidic smartphone platform for real-time detection, counting, and sizing of living algae. Reproduced with permission from ref. [29] Copyright (2022) Elsevier.

As illustrated in Figure 2 [19,20,21,22,23,24,25,26,27,28,29,30], research on the detection, analysis, and application of marine microorganisms based on microfluidic technology generally encompasses six key stages: sample capture and collection, sample separation and enrichment, microbial culture, qualitative or quantitative detection, and data inversion and application. The C-shaped and U-shaped channels designed in Figure 2a,b demonstrate enhanced capabilities in capturing microdroplets and microparticles. Figure 2c,d present various separation techniques. Figure 2e depicts the enrichment efficiency across different bacterial communities. Figure 2f,g describe the biological responses of microalgae under distinct microenvironmental conditions, including exposure to light alone and light combined with carbon dioxide. Figure 2h illustrates the formation and cultivation of a biofilm. Figure 2i,j outline the principles and implementation of fluorescence-based detection and impedance counting methods, respectively. Finally, Figure 2k,l highlight the practical applications of microfluidic systems in monitoring harmful algal blooms and assessing the physiological activity of algal microorganisms. Collectively, these six integrated technical stages have undergone substantial development and refinement, driven by continuous advancements in interdisciplinary fields such as fluid mechanics, analytical chemistry, optics, molecular biology, immunology, data science, and artificial intelligence.

Among the six key technical components mentioned above, particle separation in microfluidic channels (including both active and passive methods) and the related fluid mechanics mechanisms have long been regarded as important research challenges and core concerns. However, in microfluidic chips constructed with elastic materials such as polydimethylsiloxane (PDMS), due to the fluid–solid coupling effect, the channel structure often undergoes a morphological change from regular to irregular. This change mainly stems from the “compliance” property [32,33,34,35,36,37] inherent in elastic materials. In the research on passive separation of particles in microfluidic fluid manipulation, many experts and scholars, when exploring the separation mechanism of micro-particles driven by Newtonian fluids such as water [38], usually base their studies on the assumption of rigid walls, investigating the inertial aggregation of particles caused by shear lift force in “straight channels” [39,40,41] or the particle separation phenomenon induced by secondary flow and Dean vortices in “multi-round arc channels” [42,43,44], and have achieved significant results. However, the phenomenon that the compliance deformation of elastic materials caused by hydraulic effects leads to changes in channel geometry, thereby affecting the distribution or flow pattern of typical flow fields such as Poiseuille flow, needs to be given sufficient attention. In fact, this structural change has a substantial impact on the fluid manipulation process in microfluidic chips, especially in multi-stage series fluid pathways, where it may produce a “cumulative effect”, making the precise control of fluids more complex. In the research on bacterial separation inertial microfluidic technology based on LOC systems, Boran Zhang et al. (2022–2023) proposed a novel particle size sorting technique based on the stable expansion mechanism of viscous microfluidic sheath flow through the study of the stable expansion phenomenon of viscous microfluidic sheath flow [45,46]. Meanwhile, the team also developed a double-layer microchannel geometry with a groove array structure, achieving effective manipulation of nanoparticles [47]. Notably, this research team has also made significant contributions in the application of inertial microfluidics-assisted membrane-based microfiltration [48].

**Figure 3:**
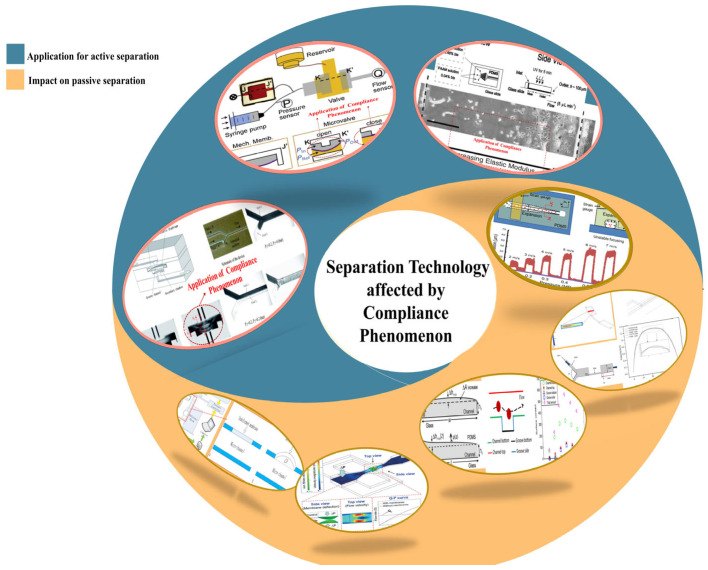
The “compliance” phenomenon in microfluidic systems. Reproduced with permission from ref. [54] Copyright (2004) WILEY-VCH [55], copyright (2019). Elsevier B.V. [56] Copyright (2012). Royal Society of Chemistry [53], copyright (2016). MDPI AG [50], copyright (2015). Royal Society of Chemistry [57], copyright (2006). Royal Society of Chemistry [52], copyright (2016). Springer Nature [49]. Copyright (2006). AIP Publishing [51], copyright (2020). WILEY-VCH.

Building upon this foundation, this paper first provides a concise overview of the application of microfluidic chip technology in the analysis of marine microorganisms. It then presents an in-depth investigation into the “compliance” phenomenon observed in typical polydimethylsiloxane (PDMS)-based microfluidic systems as illustrated in Figure 3 [49,50,51,52,53,54,55,56]. The study systematically reviews the underlying mechanisms, recent advancements, and the influence of compliance behavior in mechanically elastic materials on microfluidic performance. Furthermore, the paper examines the functional roles of compliance in key operational processes of microfluidic technology, such as the capture, separation, enrichment, and detection of microorganisms and particles. Additionally, it explores the interplay between surface wettability engineering and the compliance phenomenon. This review aims to enhance the understanding and control of the mechanical behavior of fluids and their carried particles within microfluidic systems, offering valuable theoretical insights and practical guidance to researchers in the field.

## 2. The Compliance Phenomenon of Elastic Materials in Microfluidic Systems

The compliance phenomenon of elastic materials in microfluidic systems is an important research field, involving the influence of mechanical deformation of materials on fluid flow behavior. **Compliance refers** to the ability of material to undergo reversible deformation (elastic deformation) under external force. In LOC systems, the characterization and measurement methods are associated with the degree of volume or shape change in the material under pressure. The derivation process of its mathematical equation (Timoshenko & Goodier 1970 [58] is as follows,

From Hooke’s Law in one dimension:

(1) σ=Eε

Extension to three-dimensional stress state:

(2) $$\sigma_{ij}=\lambda \delta_{ij}\varepsilon_{kk}+2\mu \varepsilon_{ij}$$

Consider the condition of hydrostatic pressure:

(3) $$\;\;\sigma_{11}=\sigma_{22}=\sigma_{33}=-P$$

Finally, it is concluded that:

(4) $$C=\frac{∆V}{∆P}$$

Here, ∆V denotes the volume change, and ∆P represents the applied pressure change.

The main factors affecting compliance include: the elastic modulus of the material, Poisson’s ratio, geometric shape, and boundary conditions. While in the time scale, the main manifestations of the compliance phenomenon include instantaneous elastic response and viscoelastic time-dependent behavior.

In the practical engineering application of LOC systems, the compliance of elastic materials causes the materials to deform under fluid pressure, which in turn affects the geometry of the channel and the flow characteristics. This phenomenon was experimentally verified by Gervais et al. (2006) [57] and further theoretically supported by Hardy et al., 2009 through finite element analysis [59]. Therefore, considering the geometric constraints within the microchannel, the compliance equation in the microchannel of the LOC system can be expressed as follows.

Consider the aspect ratio of the channel:

(5) $$f_{1}=\frac{w}{h}$$

Consider the effect of channel wall thickness:

(6) $$f_{2}=\frac{t}{w}$$

Consider the boundary conditions $f_{3}(boundary)$ of the channel, The final expression of the compliance equation in the microchannel is obtained:

(7) $$C_{channel}=C\cdot f_{1}\cdot f_{2}\cdot f_{3}\left(boundary\right)$$

From the perspective of practical application of LOC, the phenomenon that “compliance” in microfluidic systems affects the channel geometry and flow characteristics is of great significance in actual engineering applications. It may bring adverse effects or be ingeniously utilized to achieve specific functions. Subsequently, this paper will delve into the compliance issue in LOC systems and its influence on passive separation operations and summarize the latest research progress in utilizing the compliance of elastic materials.

### 2.1. Description of Compliance Issues in the Microchannels of the LOC System

As mentioned earlier, during the development and operation of the LOC system, **compliance effects** can give rise to several key engineering challenges, mainly in the following two aspects: firstly, the accuracy of fluid flow control; secondly, the dynamic response characteristics of the microchannel integrated system.

In the research on the flow control accuracy of microchannels, Gervais et al. [57] (2006) measured the actual flow rate of PDMS microchannels using high-precision flowmeters, established a channel deformation model based on the Young-Laplace equation, and further verified the theoretical prediction results of the model using the finite element analysis method. The research results show that within the pressure range of 100–1000 Pa, the flow deviation of the LOC microchannel system can reach 20% to 50%; the aspect ratio of the channel has a significant impact on compliance; therefore, it is suggested that the wall deformation factor should be comprehensively considered in flow calculation and the traditional Poiseuille equation should be modified. Oh et al. [60] (2012) designed a PDMS chip containing eight parallel microchannels and combined with fluorescence tracing technology to achieve real-time monitoring of the flow rate of each channel, systematically studying the influence of pressure pulsation frequency on the uniformity of flow distribution. The study found that in a multi-channel system, the flow non-uniformity can be as high as 30%, and the pressure pulsation within the frequency range of 1–10 Hz has the most significant impact on the flow distribution. Based on this, the researchers proposed a method to compensate for the compliance of microchannels by introducing rigid inserts.

In the study of dynamic response characteristics of microchannel integrated systems, Mosadegh et al. (2010) [61] analogized microfluidic systems to electrical circuits and defined the corresponding flow resistance equation as $R=\frac{12\mu L}{wh^{3}}$ and the compliance C was defined as Equation (4).This research team tested the response time of microchannels under different geometric parameters, further constructed an RC equivalent circuit model of the microfluidic system, and derived the expression for the response time as $t_{response}=RC$.The research results show that the response time range based on the channel geometric parameters is from 0.1 to 10 s, and it clarifies that there is a linear relationship between the pressure establishment time and compliance. Raj and Sen et al. (2016) [62] used high-speed camera technology to record the deformation process of elastic microchannels under fluid action, established a second-order system transfer function model was established to describe its dynamic response characteristics. The time-domain response characteristic equation is as follows,

(8) $$\;\;y\left(t\right)=K\left[1-e^{-\zeta \omega_{n}t}\left(cos\left(\omega_{dt}\right)+\frac{\zeta }{\sqrt{1-\zeta^{2}}}sin\left(\omega_{d}t\right)\right)\right](\zeta <1)$$

Quantitative analysis was conducted on key parameters such as the damping ratio $\zeta =\frac{\mu }{2pL}\sqrt{\frac{\rho }{E}}$ and the natural frequency $\omega_{n}=\sqrt{\frac{E}{\rho L^{2}}}$. The research found that the oscillation frequency is directly proportional to the elastic modulus of the material, as shown in the formula: f∝Eρ. During the oscillation process, the overshoot amplitude can reach 20% to 40% of the steady-state value. The time $t_{settle}$ required for the system to reach the steady state can be described by the following equation:

(9) $$t_{settle}=\frac{4}{\zeta \omega_{n}}$$

In the above equation or formula, E stands for the elastic modulus of the material (Pa), ρ stands for fluid density (kg/m^3^), L  stands for characteristic length (channel length) (m), μ stands for dynamic viscosity of the fluid (Pa·s). ζ corresponds to the damping ratio, and $\omega_{n}$ corresponds to the natural frequency.

Other research teams, such as Hardy et al. (2009) [59], have studied the long-term impact of PDMS aging on compliance from the perspective of a single material property and found that its elastic modulus increases over time, thereby affecting system stability. Cheung et al. (2012) [63] developed real-time compliance monitoring technology and independently proposed an adaptive flow control algorithm, improving the flow control accuracy within the microchannels of the LOC system to within ±5%.

Henrik Bruus et al. (2008) [64] described the phenomenon of “compliance” in LOC systems in a summary form in their work. This description analogizes microchannel systems to circuit models based on the mathematical similarity between Hagen–Poiseuille’s law and Ohm’s law. This analogy implies that in LOC systems, the pressure drop ∆p within the microchannel and the volumetric flow rate Q (∆V,volume flowing per unit time) can, respectively, correspond to the voltage drop ∆U and the current I (charge q flowing per unit time) in a circuit system.

It is well known that the capacitance $C_{E}$ can be expressed by formula CE=dqdU. Similarly, in fluid systems, the “hydraulic capacitance C” has been introduced, also known as compliance. However, it should be noted that the hydraulic capacitance C and the capacitance $C_{E}\;$ have different physical meanings and can even be considered opposite. This is because in microchannels, the volume of the fluid usually decreases as the pressure increases. In LOC microchannel systems composed of elastic materials such as PDMS, due to the elastic properties of the material itself, the actual closed chamber or channel where the fluid is located cannot be completely rigid. Therefore, the volume of the system will change accordingly with the variation in external or internal pressure, although this change is usually very small.

**Figure 4:**
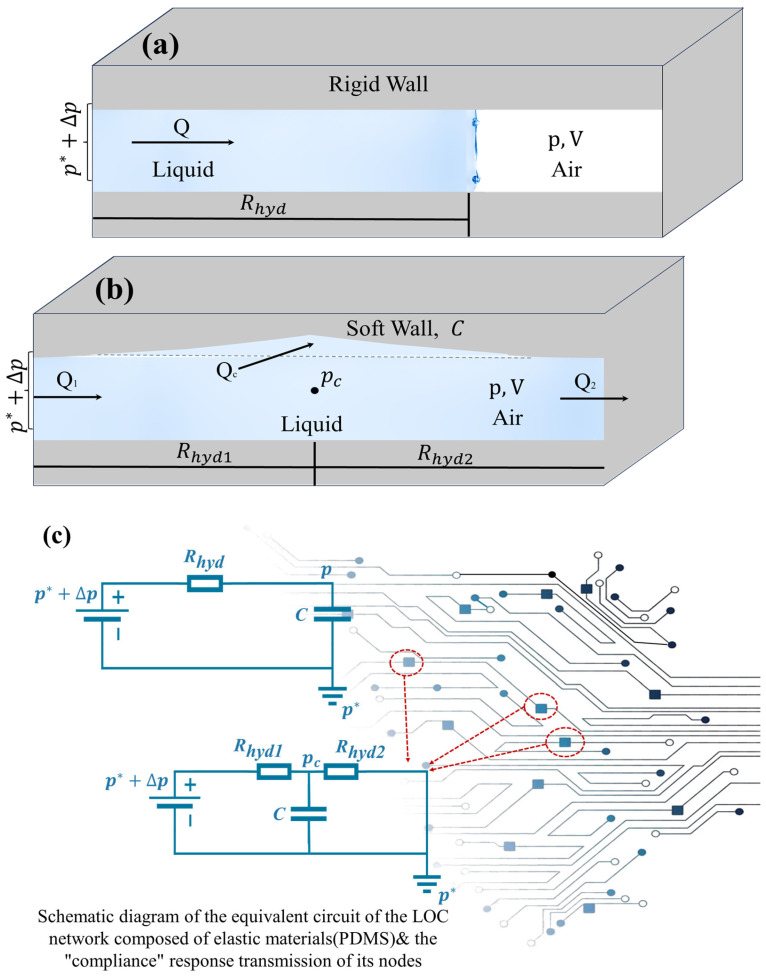
Schematic diagram of compliance contrast and equivalent circuit of LOC network and its node response transfer. (**a**) Schematic diagram explaining the mechanism of compliance in rigid-wall conditions. (**b**) Schematic Diagram Explaining the Mechanism of Compliance in Soft-Wall Conditions. (**c**) Schematic of the equivalent circuit for a LOC network composed of elastic material (PDMS) and its node “compliant” response transmission.

As illustrated in Figure 4a, a simplified example is presented to demonstrate the compliance of gas confined within a channel under the assumption of rigid walls. This example describes the volume of gas trapped within a closed channel when a liquid advances and acts as a liquid piston. Compared to the liquid and the channel walls, the gas exhibits significantly higher compliance. Initially, at atmospheric pressure $\;p^{*}$, the gas occupies a volume $\;V^{*}$. However, as the liquid progresses into the channel, both the gas volume V  and pressure p change accordingly. Assuming an isothermal process, the ideal gas law can be applied $pV\;=\;p^{*}V^{*}$. Based on this relationship, the hydraulic compliance is derived as C=p∗V∗p2. When the pressure variation is relatively small compared to $\;p^{*}$, the gas compliance simplifies to  C≈V∗p∗. At time t < 0, the pressure throughout the system remains at $p^{*}$ At t = 0, the pressure at the liquid inlet is abruptly increased from $p^{*}$ to $p^{*}+∆p$. Consequently, the liquid begins to advance at a volumetric flow rate Q(t), leading to a rapid reduction in the gas volume. Applying the chain rule, the flow rate can be expressed as:

(10) $$Q\left(t\right)=-\partial_{t}V=-\left(\partial_{p}V\right)\partial_{t}p=\;C\partial_{t}p\;\;$$

According to the Hagen–Poiseuille law, the pressure drops across the channel is related to the flow rate by:

(11) $$\left(p^{*}+∆p\right)-p=R_{hyd}Q=-R_{hyd}\partial_{t}V=R_{hyd}C\partial_{t}p$$

Here, $R_{hyd}$ can be analogously regarded as resistance.

Therefore, the solution for the gas pressure *p*(*t*) existing at this time can be easily seen as similar to the voltage on a charging capacitor with characteristic RC time *t*, given by the equation:

(12)   pt=p∗+1−e−tτ∆p

where $\tau \equiv R_{hyd}C$.

As shown in Figure 4b, as an example of fluid compliance, we consider a model of a soft-walled channel filled with incompressible fluid. If the pressure inside the channel increases, the channel will expand and deform. Suppose the compliance C of the channel is a given constant, which is related to the geometry and material properties of the channel wall. For simplicity, we model the channel as consisting of two sub-channels with hydraulic resistances $R_{hyd1}$ and $R_{hyd2}$ connected in series. The pressure $p_{c}$ at the junction of the two sub-channels determines the expansion of the entire channel. As mentioned earlier, we assume that the inlet pressure is $p^{*}$ for t < 0 and $p^{*}+∆p$ for t > 0. The inlet and outlet flow rates are $\;Q_{1}=\;(p^{*}+∆p-p_{c})\;/\;R_{hyd1}$ and $Q_{2}=\;(p_{c}-p^{*})\;/R_{hyd2}$, respectively, and the volume expansion rate of the cavity is

(13) $$Q_{c}=-\partial_{t}V=C\partial_{t}p_{c}$$

Since the fluid is assumed to be incompressible, by mass conservation, we have $Q_{1}$ = $Q_{2}$ + $\;Q_{c}$, and we obtain the differential equation for the pressure pc inside the channel as follows,

(14) $$\partial_{t}p_{c}=-\left(\frac{1}{\tau_{1}}+\frac{1}{\tau_{2}}\right)p_{c}+\left(\frac{1}{\tau_{1}}+\frac{1}{\tau_{2}}\right)p^{*}+\frac{1}{\tau_{1}}∆p$$

where $\tau_{1}$ = $R_{hyd1}C$ and $\tau_{2}$ = $R_{hyd2}\;C$ are the hydraulic RC times.

Its solution $p_{c}\left(t\right)\;$ equation,

(15) $$\;\;p_{c}\left(t\right)=p^{*}+\left(1-e^{-\left[{\tau_{1}}^{-1}+{\tau_{2}}^{-1}\right]t}\right)\frac{\tau_{2}}{\tau_{1}+\tau_{2}}∆p$$

which quantitatively describes the compliance C of the channel as similar to the voltage on a capacitor being charged through a voltage divider. Thus, considering that the deformation of the flexible channel is affected by the hydraulic pressure, the formula $R=\frac{12\mu L}{wh^{3}}$ described in the previous text needs to be modified to $R_{eff}\;=\frac{12\mu L}{w{\left(h+\delta \left(P\right)\right)}^{3}}$, where $\delta \left(P\right)$ is the pressure-related deformation.

As shown in Figure 4c, it is the equivalent circuit of the LOC network composed of elastic materials such as PDMS and the schematic diagram of the “compliance” response transmission at its nodes. The upper and lower parts of the figure correspond to the structures shown in Figure 4a,b, respectively. In the upper figure, $R_{hyd}$ represents the hydraulic resistance of the liquid part in the channel, and  C≈V∗p∗  represents the compliance of the trapped air. The lower figure is the equivalent circuit corresponding to the flexible channel in Figure 4b, where $R_{hyd1}$ and $R_{hyd2}$ are the hydraulic resistances of each part of the channel, and C is the compliance of the soft wall, which has mentioned above. Due to the introduction of the analogy relationship between Hagen–Poiseuille’s law and Ohm’s law, the mature circuit theory methods can be applied to the microfluidic network of the lab-on-a-chip (LOC) system in a similar way to the series/parallel connection of resistors and capacitors, which has significant advantages. Therefore, for the more complex microfluidic LOC networks, their equivalent circuit models can be drawn, and based on the micro-nano fluid mechanics mechanism, they can be reasonably analyzed by analogy with Kirchhoff’s laws.

Similarly, in LOC systems with multiple flexible flow channels, based on the above analogical derivation process and drawing on the combination rules of resistors and capacitors in series/parallel coupling, applying the classic methods of circuit theory to the modeling of microfluidic networks has significant advantages. Thus, it can be concluded that for any given microfluidic network, an equivalent circuit model can be constructed. In this model, channels with hydraulic resistance $R_{hyd}$ correspond to resistors, channels with hydraulic compliance C correspond to capacitors, volumetric flow rate Q corresponds to current, and a pump providing a constant pressure difference ∆p corresponds to a battery.

For any given fluid network or its equivalent circuit model, Kirchhoff’s laws can be applied if the following two basic conditions are met:

(a) The total flow into or out of any node in a circuit is zero (the principle of conservation of mass);
(b) The algebraic sum of all pressure differences within any closed loop in a circuit is zero (the principle of conservation of energy).

### 2.2. Influence of Microchannel Compliance on Passive Separation in Microfluidic Systems

**Figure 5:**
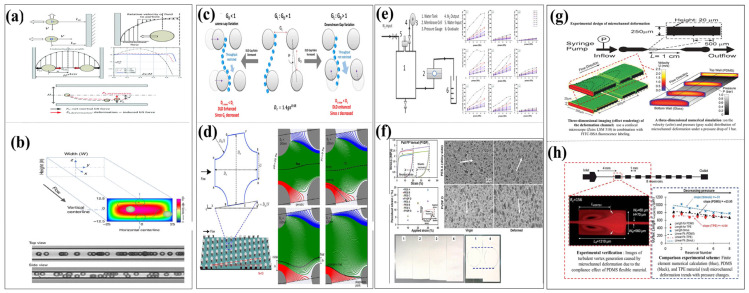
The influence of compliance in separation operations. (**a**) Mechanism diagram of inertial microfluidics. Reproduced with permission from ref. [65] Copyright (2015) Royal Society of Chemistry. (**b**) Inertial migration in a microfluidic channel cross-section visualized by high-speed imaging. Reproduced with permission from ref. [66] Copyright (2020) Springer Nature. (**c**) Effect of column gap variation on DLD micro-particle separation. Reproduced with permission from ref. [67] Copyright (2016) Springer Nature. (**d**) Dynamical map of small particles in DLD arrays under fluid symmetry breaking. Reproduced with permission from ref. [68] Copyright (2017) PNAS. (**e**) Compressibility and stability characterization of microfiltration membranes. Reproduced with permission from ref. [69] Copyright (2015) Elsevier B.V. (**f**) Mechanical property characterization of polymer microfiltration membranes. Reproduced with permission from ref. [70] Copyright (2019) Elsevier B.V. (**g**) Experimental design, imaging, and CFD analysis of flow-induced deformation in shallow microfluidic channels. Reproduced with permission from ref. [57] Copyright (2006) Royal Society of Chemistry. (**h**) Compliance deformation experiment design and microscopic characterization in microfluidic devices. Reproduced with permission from ref. [71] Copyright (2011) Royal Society of Chemistry.

As shown in Figure 5, the compliance phenomenon has a significant impact on the sample separation operation, which is crucial to the functional application of the lab-on-a-chip (LOC) system. This paper mainly focuses on the analysis of the passive separation operation mechanism influenced by fluid dynamics, with a particular emphasis on typical applications such as inertial focusing, DLD (Deterministic Lateral Displacement), microfiltration and sieving.

As shown in Figure 5a, in the study of the influence of the compliance of elastic materials on the inertial focusing of particles, Zhang et al. (2016) [65] and Zhou et al. (2020) [66] in their research and reviews, used PDMS materials with different elastic moduli, under various flow rates and pressure conditions, to record the particle movement trajectories using high-speed microscopic imaging technology and obtain the flow field distribution through micro-particle image velocimetry (μPIV). Meanwhile, the researchers measured the deformation of the microchannel and the resulting fluid pressure changes through pressure or optical devices. By combining finite element method simulations of channel deformation, computational fluid dynamics (CFD) analysis of flow field changes, and particle dynamics models to study the force and motion behavior of particles, they systematically analyzed the mechanism by which channel deformation affects the equilibrium position of particles. The research shows that channel deformation can lead to a 15–30% decrease in particle focusing efficiency, a shift in the focusing position, and reveals the quantitative relationship between deformation and focusing performance, providing a basis for establishing a theoretical system of “deformation-flow field-particle motion”.

In the analysis of the hydrodynamic mechanism where the compliance of elastic materials such as PDMS causes microchannel deformation and thereby affects particle inertial focusing, Segré & Silberberg (1961) [72] first discovered the inertial focusing phenomenon of particles in pipes. Over the subsequent 50-plus years, Ho & Leal (1974) [73] and Asmolov (1999) [74] established and refined the theory of inertial lift in inertial focusing, and Di Carlo et al. (2007) [75], Bhagat et al. (2008) [76] and Zhou & Papautsky (2013) [77] introduced the inertial focusing theory into microchannels. The hydrodynamic mechanism of inertial lift lies in the inertial lift force acting on the particles, and its generation mechanism is based on the following hydrodynamic principles: inertial lift originates from the nonlinear inertial effect of the flow field around the particles, and the total inertial lift equation,

(16) $${\vec{F}}_{L}={\vec{F}}_{shear}+{\vec{F}}_{wall}$$

Shear-induced lift (${\vec{F}}_{shear}$) and wall-induced lift (${\vec{F}}_{wall}$) are included. Among them, ${\vec{F}}_{shear}$ is the lift force generated by particles in shear flow, also known as Saffman lift (at low Reynolds numbers), and its equation is as follows,

The Saffman force is given by:

(17) $${\;\;\;\;\;\;\;\;\;F}_{Saffman}=1.615\mu \alpha^{2}\sqrt{\dot{\frac{\gamma \rho }{\mu }}}\cdot \left(U_{f}-U_{p}\right)$$

Here, μ-fluid viscosity, α-particle radius, $\dot{\gamma }$-shear rate, $U_{f}$, $U_{p}$-fluid and particle velocities.

The wall-induced lift force ${\vec{F}}_{wall}$ is the lift effect produced by the constraint of the flow field by the channel wall when the particles approach the wall surface. Its general form equation is

(18) Fwall=ρUm2a4CwallRep,ya,ha

Here, $C_{wall}$ is the wall lift coefficient, ya is the dimensionless distance of the particle from the wall, and ha is the ratio of the channel height to the particle diameter. The precise expression of the lift coefficient is

(19)    Cwall=Rep216⋅Gya,ha

Although the direction of the ${\vec{F}}_{wall}$ always points towards the center of the channel, when ya→0, the ${\vec{F}}_{wall}$ shows a repulsive effect; when ya→h2a , the ${\vec{F}}_{wall}\to 0$ at the center of the channel. Therefore, it is not difficult to see that the intensity of the wall-induced lift force is closely related to the ratio of the channel size to the particle size. Thus, the constraint parameter β=ah  is introduced to measure the strength relationship between the ${\vec{F}}_{shear}$ and the ${\vec{F}}_{wall}$ due to the scale effect, that is:

- β<0.1: The wall effect is weak, and the shear lift force is dominant.
- 0.1 < β<0.3: The wall effect is significant and affects the equilibrium position.
- β>0.3: Strong constraint condition, the wall effect is dominant.

In conclusion, the modified equation for the wall-induced lift force ${\vec{F}}_{wall}$ is obtained.

(20) Fwall=ρUm2a4Rep216⋅β2⋅fyh

Here, fyh is a position-dependent function.

**In the microchannel application of the LOC system, due to the flow being within the moderate Reynolds number range**, McLaughlin (1991) [78] provided a complete expression for the inertial lift at finite Reynolds numbers, namely the McLaughlin correction equation,

(21) $$\;F_{L}=\rho U_{m}^{2}a^{4}C_{L}\left({Re}_{p},\kappa \right)$$

Here, Rep=ρUmaμ is defined as the particle Reynolds number, and κ=ah represents the ratio of particle size to channel size. However, it is particularly important to note that the $C_{L}$ here is the lift coefficient function within the microchannel at moderate Reynolds numbers, and its empirical equation is

(22) $$C_{L}=\frac{3}{16\pi^{2}}{{Re}_{p}}^{2}\kappa^{4}f\left(\kappa \right)$$

The compliance phenomenon of elastic materials such as PDMS causes microchannel deformation and exists in various geometric deformation mechanisms of the channel, including:

- The expansion and deformation of rectangular channels under pressure;
- An increase in channel height $h_{eff}=h_{0}+\delta$;
- A slight increase in channel width, leading to a transformation of the cross-sectional shape from rectangular to elliptical, etc.

By introducing the theory of elasticity, the deformation of PDMS channels can be expressed by the equation,

(23) $$\;\delta =\frac{∆P\cdot h_{0}^{3}}{12E\cdot d}\cdot f\left(\nu ,geometry\right)$$

Here, E represents the elastic modulus of PDMS, d is the thickness of the top of the channel, and ν is the Poisson’s ratio.

At the same time, the deformation of micro-channels will cause changes in the flow field characteristics, resulting in variations in the effective Reynolds number.

(24) $${Re}_{eff}=\frac{\rho U_{max}D_{h}}{\mu }\cdot \frac{h_{eff}}{h_{0}}$$

The maximum flow velocity position is displaced, and the shear rate distribution changes. The secondary flow intensity is affected by the pressure gradient in the deformed channel, which is given by,

(25) $$\;\frac{dP}{dx}=-\frac{12\mu Q}{\omega h_{eff}^{3}}\cdot {\left(1+\frac{\delta }{h_{0}}\right)}^{-3}$$

Therefore, integrating the research of Gossett & Di Carlo (2009) [79], Hur et al. (2011) [80], and Masaeli et al. (2012) [81], the influence of deformation caused by compliance on the particle focusing position and flow field distribution within microchannels was investigated, and the inertial focusing model was revised. Thus, after considering the inertial lift correction due to PDMS deformation, an inertial focusing model equation with a deformation correction factor was established.

(26)  FL=ρUm2a4CLRep,κeff⋅Φδh

Here, κeff=aheff represents the effective size ratio, $h_{eff}$ is the effective channel height, and Φδh is the deformation correction factor. The empirical relationship of the deformation correction factor is given as

(27)  Φδh=1+aδh+βδh2

As vividly illustrated in Figure 5c,d, this section delves into the profound impact of mechanical compliance in flexible materials on the evolution and performance of Deterministic Lateral Displacement (DLD) technology within the realm of microfluidics. The pioneering work of Huang et al. (2004) [82] laid the cornerstone for DLD technology by introducing its foundational concept and constructing a theoretical framework rooted in rigid structural principles. Building upon this foundation, McGrath et al. (2014) [83] contributed a comprehensive and insightful review that meticulously evaluated the development and integration of DLD technology within Lab-on-a-Chip (LOC) systems. Further enriching the field, Bowman et al. (2012) [84] conducted in-depth investigations into the separation dynamics of force-driven droplets within DLD arrays, while Christov (2021) [85] offered a rigorous and systematic exposition of the theoretical underpinnings of flexible microfluidic systems.

As computational capabilities have advanced alongside technological progress, the synergy between numerical computation, CFD simulations, and theoretical modeling has significantly propelled both the understanding and application of DLD technology. Krüger et al. (2010) [86] employed advanced three-dimensional simulations to explore the intricate deformation and separation behaviors of red blood cells within DLD systems. Continuing this line of inquiry, Chien et al. (2019) [87] meticulously examined how the dynamic deformability of red blood cells influences their traversal behavior in DLD-based microstructures, thereby expanding the frontiers of biomedical microfluidics.

Through in-depth research on the above-mentioned research results, Ye et al. (2014) [88] described the quantitative relationship of critical diameter variation between elastic particles and the distance of DLD columns, and this nonlinear relationship can be described by the following mathematical model:

(28) $$D_{c}=D_{c0}\cdot f\left(\kappa ,Re,\lambda \right)$$

From the perspective of quantifying the flow field distribution, their research results also indicate that an increase in the distance between columns has a significant impact on the separation performance. Changes in both the longitudinal and lateral spacings will alter the critical particle size, $D_{c}$.

Using the modified form of the Davis empirical formula:

(29) $$D_{c}=1.4\cdot G_{eff}\cdot \varepsilon^{0.48}$$

Here, $D_{c}$ is the critical separation diameter, $G_{eff}$ is the effective column spacing after considering deformation, and ε is the shift fraction.

The calculation equation for the effective column spacing is

(30) $$G_{eff}=G_{0}\left(1-\delta_{compliance}\right)$$

Here, $\delta_{compliance}$ represents the relative deformation caused by material compliance.

Regarding the correction for the variation in anisotropic permeability, we have,

(31) $$AP=\frac{∆p_{x}}{∆p_{y}}$$

Here, $∆p_{x}$ transverse pressure gradient, $∆p_{y}$ longitudinal pressure gradient

For flexible systems with compliance effects, the modified AP equation is

(32) $$\;{AP}_{compliance}={AP}_{0}\cdot \left(1+\alpha \cdot \frac{∆P}{E_{material}}\right)$$

Among them:

- ${AP}_{0}$: Anisotropic permeability of the rigid system;
- α: Material-related correction coefficient;
- ∆P: Delta P$—System pressure difference;
- $E_{material}$: Elastic modulus of the material.

The above-mentioned influences are reflected in the evaluation indicators of the separation efficiency of the LOC system. For the separation of biological particles, McGrath et al. (2014) [83], Dincau et al. (2018) [89], and Brian Senf & Jong-Hoon Kim (2022) [90] quantified the impact of compliance on DLD technology through the following three indicators:

Separation resolution: Achieving a separation accuracy of up to 10 nm;

Processing throughput: Enhanced separation performance under high Reynolds number conditions;

Viscosity effect: Quantitative influence of viscosity changes on high-throughput DLD performance.

During the DLD separation operation in the LOC system, the main principle followed is the dynamic nature of fluid–structure interaction based on the mechanical compliance of flexible materials. Therefore, in the flexible DLD device, the mathematical description of fluid–structure interaction (FSI) requires the coupling of fluid dynamics equations and structural mechanics equations. Thus, within the smooth operation area of DLD, there exists control equations for the fluid domain.

(33) $$∆\cdot u=\rho_{f}\frac{\partial u}{\partial t}+\rho_{f}\left(u\cdot \nabla \right)u=-\nabla p+\mu \nabla^{2}u$$

Domain control equation,

(34) $$\rho_{s}\frac{\partial^{2}d}{\partial t^{2}}=\nabla \cdot \sigma +f_{s}$$

Here, u represents the fluid velocity field, p the pressure field, d the structural displacement, σ the stress tensor, $\rho_{f}$ and $\rho_{s}$ the densities of the fluid and solid, respectively, μ the dynamic viscosity, and $f_{s}\;$ the volume force.

The existence of the control equations is ensured when the boundary conditions at the fluid–solid interface and the coupling conditions are incorporated to make the equations closed.

The kinematic no-slip condition,

(35) $$u_{f}\left|\Gamma =\frac{\partial d_{s}}{\partial t}\right|\Gamma$$

Kinetic stress continuity,

(36) $$\sigma_{f}\cdot n=\sigma_{s}\cdot n$$

Fluid stress tensor:

(37) $$\sigma_{f}=-pΙ+\mu \left(\nabla u+{\left(\nabla u\right)}^{Τ}\right)$$

Here, Γ Gamma represents the fluid–structure interface, n is the normal vector of the interface, and Ι is the unit tensor. These boundary conditions ensure the continuity of velocity and stress balance at the interface between the fluid and the structure.

Based on the theoretical foundation of the DLD operation in the microchannel of the aforementioned LOC system, and referring to Mushara et al. (2024) [91] conducted a review study on fluid–structure interaction (FSI) in the field of microfluidics, focusing on the moving boundary problem caused by channel elastic deformation in microfluidic systems—that is, the impact of microchannel deformation on the flow field area and the spacing of DLD columns. The study employed the Arbitrary Lagrangian–Eulerian (ALE) method for modeling and analysis. This method can effectively handle geometric changes due to structural deformation, thereby achieving numerical simulation of fluid–structure coupling behavior with reasonable accuracy. The description of the (ALE) method is as follows,

Navier–Stokes equations in ALE coordinate system,

(38) $$\;\frac{\partial u}{\partial t}|\hat{x}+\left(\left(u-w\right)\cdot \nabla \right)u=-\frac{1}{\rho_{f}}\nabla p+\nu \nabla^{2}u$$

Grid Speed w,

(39) $$w=\frac{\partial x}{\partial t}|\hat{x}$$

Geometric Conservation Law,

(40) $$\frac{\partial J}{\partial t}+\nabla \cdot \left(Jw\right)=0$$

Here, w represents the grid velocity, J is the Jacobian determinant, $\hat{x}$ denotes the reference coordinate, ν=μρf is the kinematic viscosity.

As previously described, the Arbitrary Lagrangian–Eulerian (ALE) method allows the mesh to follow the structural deformation while maintaining the computational accuracy of the fluid domain, which enables this method to achieve a certain improvement in accuracy when studying the compliance phenomenon of elastic materials such as PDMS in the deterministic lateral displacement (DLD) technology of LOC systems. This advantage is manifested in comparison with the traditional rigid DLD critical diameter model at the inter-column effect level (critical diameter model).

According to the Inglis theoretical model (Pariset et al., 2017) [92], Small 13.37 states that,

(41) $$D_{c}=2Gsin\left(\theta \right)$$

Huang et al. (2004) [82] considered the DLD technology under the condition of uniform gap sizes between spherical particles, cylindrical columns, and all adjacent columns (without considering the influence of elastic deformation), and introduced the Davis empirical formula:

(42) $$\frac{D_{c}}{D_{y}}=1.4\varepsilon^{0.48}$$

The Unified Theoretical Framework Proposed by Kim et al. (2017) [68]

(43) $$tan\left(\theta_{eff}\right)=\frac{\varepsilon N_{eff}}{1+\varepsilon (N_{eff}-N_{p})}$$

Here, $D_{c}$ is the critical diameter, G is the column spacing, θ is the streamline deflection angle, ε is the row deflection fraction, $N_{eff}$ is the effective periodicity, $N_{p}$ is the structural periodicity, and $D_{y}$ is the lateral gap.

Since the above-mentioned models assume rigid structures and do not consider the deformation effects caused by compliance, a modified model under the influence of compliance is introduced. The effective geometric parameters are corrected, there exists an effective column spacing,

(44) $$G_{eff}=G_{0}+\Delta G\left(p,u\right)$$

The effective deflection angle of the particles is set as

(45) $$\theta_{eff}=arctan\left(\frac{\varepsilon_{eff}\lambda_{eff}}{D_{eff}}\right)$$

The corrected critical diameter of the controlled particles is

(46) $$\;\;D_{c,eff}=2G_{eff}sin\left(\theta_{eff}\right)\cdot C_{compliace}\;$$

Among them, the compliance correction factor, $\left(44\right)$ them, the compliance correction factor,

(47) $$C_{compliance}=1+\alpha \frac{\Delta p}{E}{\left(\frac{h}{G_{0}}\right)}^{\beta }$$

Here, ΔG represents the pressure-induced pore size change, Δp represents the transmembrane pressure difference, E is the material’s elastic modulus, h is the channel height, α and β are empirical constants, and $C_{compliance}$ is compliance correction factor.

To further describe the quantitative coupling relationship between the pressure field and channel deformation, the theory of elasticity mechanics is applied, considering the linear elastic approximation.

(48) $$∆G=\frac{∆p\cdot G_{0}^{3}}{12\mu QL}\cdot \frac{1}{E}{\left(\frac{h}{\omega }\right)}^{3}$$

Introduce the nonlinear large deformation model,

(49) $$∆G=G_{0}\left[{\left(1+\frac{∆p}{E_{eff}}\right)}^{\frac{1}{3}}-1\right]$$

Among them, the effective elastic modulus is given by the equation

(50) $$E_{eff}=E\cdot f\left(v,geometry\right)$$

where the geometric factor function is defined as

(51) $$\;\;\;\;\;f\left(v,geometry\right)=\frac{1-v^{2}}{1-v}\cdot {\left(\frac{\omega }{h}\right)}^{0.5}$$

Here, Q represents the flow rate, L the channel length, ω the channel width, v the Poisson’s ratio, and $E_{eff}$ the effective elastic modulus.

Simultaneously, the phenomenon of Compliance exerts a profound influence on the distribution of the flow field during DLD operation. The dynamic deformation of the flexible microchannel dramatically reshapes the hydrodynamic environment within the DLD device, thereby modulating the efficiency and behavior of particle separation.

The deformation of the flexible channel will lead to the redistribution of the velocity field, thus forming a non-uniform velocity profile in the deformed area. Based on the modified Poiseuille flow model, its velocity distribution can be expressed as:

(52) $$u\left(y,z\right)=\frac{\Delta p}{2\mu L}\left[\begin{array}{c} \frac{h_{eff}(y{)}^{2}}{4}-z^{2} \end{array}\right]$$

Among them, the effective channel height is defined as:

(53) $$h_{eff}\left(y\right)=h_{0}+\delta h\left(y,p\right)$$

The height variation function is given by the following formula,

(54) $$\delta h\left(y,p\right)=\frac{\Delta p}{E}\left(\frac{h_{0}}{2}\right)\left[\begin{array}{c} 1-\cos\left(\frac{2\pi y}{\lambda }\right) \end{array}\right]$$

Due to the change in the channel’s geometric structure, the fluid flow rate is redistributed among different channels, and its expression is

(55) $$Q_{lane,i}=Q_{total}\cdot \frac{\int_{y_{i}}^{y_{i+1}}\;h_{eff}(y{)}^{3}dy}{\int_{0}^{W}\;h_{eff}(y{)}^{3}dy}$$

Herein,

- $h_{eff}\left(y\right)$ represents the effective channel height along the transverse position y,
- δh denotes the variation in channel height,
- λ is the periodic spacing of the column array,
- $Q_{lane,i}$ indicates the flow distribution of the i-th channel,
- W is the total width of the channel.

The streamline offset phenomenon has significant dynamic characteristics, and its evolution process can be described by the following equation:

The dynamic streamline equation is:

(56) $$\frac{dy}{dx}=\frac{v\left(x,y,t\right)}{u\left(x,y,t\right)}=\tan\left(\theta_{local}\left(x,y,t\right)\right)$$

Among them, the local streamline deflection angle varies with time, and the expression is:

(57) $$\theta_{local}\left(x,y,t\right)=\theta_{0}+\Delta \theta \left(p\left(t\right),d\left(t\right)\right)$$

The influence of pressure pulsation on the offset angle can be quantified by the following relationship,

(58) $$\Delta \theta \left(t\right)=\frac{\partial \theta }{\partial p}\Delta p\left(t\right)+\frac{\partial \theta }{\partial G}\frac{\partial G}{\partial p}\Delta p\left(t\right)$$

In addition, the width of the streamline also shows a time-dependent variation characteristic, and its dynamic evolution can be expressed as:

(59) $$w_{stream,i}\left(t\right)=w_{stream,0}\left[1+\gamma \frac{\Delta p\left(t\right)}{E}\sin\left(\frac{2\pi i}{N}\right)\right]$$

Herein:

- $\theta_{local}$ represents the local streamline deflection angle,
- Δθ represents the angle change caused by structural deformation,
- $w_{stream,i}$ represents the width of the i-th streamline,
- γ is the sensitivity coefficient of streamline width to pressure variation,
- N is the total number of periods in the column array.

The deformation of flexible channels not only triggers the reconstruction of the velocity field but also induces significant vortex and secondary flow effects, further influencing the fluid dynamics behavior and particle transport characteristics. The relevant physical quantities can be described by the following theoretical model:

The modified expression of the Dean number is

(60) $$De_{eff}=De_{0}\left(1+\beta \frac{\Delta h}{h_{0}}\right)$$

Among them, the effective Dean number $De_{eff}$ considers the enhancing effect of the channel height variation on the inertial centrifugal force effect, and β is the geometric correction coefficient.

The intensity of the secondary flow is in the following proportional relationship with the main flow,

(61) $$\left|u_{secondary}\right|=\frac{De_{eff}^{2}}{Re}\cdot \left|u_{primary}\right|$$

This relationship indicates that the intensity of the secondary flow depends on the ratio of the effective Dean number to the Reynolds number Re, reflecting the competitive mechanism between the inertial and viscous effects of the flow.

The vorticity evolution process is described by the following governing equations,

(62) $$\;\frac{\partial \omega }{\partial t}+\left(u\cdot \nabla \right)\omega =\left(\omega \cdot \nabla \right)u+\nu \nabla^{2}\omega$$

Among them, the vorticity source term induced by deformation can be expressed as

(63) $$S_{\omega }=\frac{\partial }{\partial t}\left(\begin{array}{c} \nabla \times \frac{\partial d}{\partial t} \end{array}\right)$$

This source term reveals the contribution mechanism of the structural deformation rate to vorticity generation.

In summary, the above-mentioned eddy current and secondary flow effects are particularly prominent in flexible channels, exerting a significant influence on the lateral migration behavior of particles and thereby regulating the separation and manipulation performance in microfluidic systems.

As shown in Figure 5e,f, the compliance effect has a significant impact on the size-based sieving and separation behavior during the microfiltration and sieving operations in LOC (Lab-on-a-Chip) systems. The dynamic change in pore size, as a direct manifestation of the compliance effect, significantly influences the separation performance of the system. In practical applications, microfiltration and sieving operations typically rely on a “membrane” as a functional structure, and compliance affects the separation process by influencing the compaction behavior of the membrane structure and the mechanical properties of its material. Yu Han et al. (2015) [69] investigated the membrane compaction behavior of polysulfone (PS), polyethersulfone (PES), and polyvinylidene fluoride (PVDF) flat sheet membranes during pressure-driven filtration and clarified the influence mechanism of compliance on filtration performance. The study indicated that the reduction in residual deformation and the improvement of membrane structural stability suggest that elastic deformation plays a significant role in the long-term operational performance of the membrane.

Elele et al. (2019) [70] systematically investigated the relationship between the mechanical properties of membrane materials and the pore topology by analyzing the influence of pore topology and polymer properties on the mechanical performance of asymmetric polyethersulfone (PES) and symmetric polyvinylidene fluoride (PVDF) microfiltration membranes. This study provides a theoretical basis for the prediction and design of mechanical properties of membranes made of different elastic materials, which is helpful for optimizing the selection and application of membrane materials. Elele et al. [70] systematically investigated the mechanical properties of polymeric microfiltration membranes, providing experimental verification and theoretical support for the theory of pore expansion in elastic membranes under pressure. The study demonstrated that the viscoelastic behavior of membrane materials has a significant impact on filtration performance.

For membranes made of elastic materials, the pore radius will expand under the action of internal pressure. This expansion process competes with the particle deposition process, thereby delaying the occurrence of membrane fouling and further affecting the overall filtration performance. Specifically, the dynamic balance between pore expansion and particle deposition determines the evolution characteristics of filtration efficiency over time. According to the theory of elasticity, under the basic assumptions that the membrane material is a linear elastic body, the pore is circular, the deformation is small, and the stress in the thickness direction of the membrane is ignored, the radius change in the elastic membrane pore under the transmembrane pressure ΔP can be expressed as:

(64) $$r_{eff}=r_{0}\left(1+\epsilon_{r}\right)$$

Here, $r_{eff}$ represents the effective aperture radius, $r_{0}$ is the initial aperture radius, and $\epsilon_{r}$ is the radial strain.

Furthermore, for thin film structures, the relationship between radial strain and transmembrane pressure can be expressed by the following equation,

(65) $$\epsilon_{r}=\frac{\Delta P\cdot r_{0}}{E\cdot t}\cdot \left(1-\nu \right)$$

Here, E represents the elastic modulus of the membrane material (Pa), t is the membrane thickness (m), ν is the Poisson’s ratio, and ΔP is the transmembrane pressure difference (Pa). From the above formula, the degree of pore expansion is directly proportional to the transmembrane pressure and inversely proportional to the material stiffness (i.e., E). Additionally, Poisson’s ratio ν reflects the lateral contraction effect of the material under force.

Therefore, the complete expression for the effective aperture is:

(66) $$\;\;r_{eff}=r_{0}\left[1+\frac{\Delta P\cdot r_{0}\cdot \left(1-\nu \right)}{E\cdot t}\right]$$

If the flexibility parameter $C_{f}=\frac{r_{0}(1-\nu )}{E\cdot t}$ is introduced, the above equation can be simplified to:

(67) $$r_{eff}=r_{0}\left(1+C_{f}\cdot \Delta P\right)$$

This simplified model provides a theoretical basis for analyzing the pore size changes in elastic membranes under pressure-driven conditions and is helpful for understanding the influence mechanism of compliance on the microfiltration process.

From the above simplified form, the larger the value of the compliance parameter $C_{f}$, the softer the membrane material is, and the more significant the pore expansion effect under pressure. The magnitude of $C_{f}$ is determined by the intrinsic properties of the membrane material and its geometric structure. In engineering practice, even a slight expansion of the pore diameter can significantly improve the permeation performance of the fluid. The physical mechanism of this phenomenon lies in the quartic relationship between the effective permeability $K_{eff}$ and the pore radius, which amplifies the impact of the compliance effect on the system performance.

According to the Hagen-Poiseuille equation, the permeability of a circular channel is proportional to the fourth power of the radius of the aperture, that is,

(68) $$K\propto r^{4}$$

Therefore, after considering the aperture expansion effect, the effective permeability of the system can be expressed as

(69) $$K_{eff}=K_{0}\cdot {\left(\frac{r_{eff}}{r_{0}}\right)}^{4}=K_{0}(1+C_{f}\cdot \Delta P{)}^{4}\;$$

Under the small deformation approximation condition (i.e., $C_{f}\cdot \Delta P\ll 1$), a Taylor expansion of the above equation yields the approximate expression,

(70) $$K_{eff}\approx K_{0}\left(1+4C\cdot \Delta P\right)$$

This approximate model clearly reveals the linear enhancement effect of compliance parameter $C_{f}$ and pressure change ΔP on permeability performance, providing a theoretical basis for engineering design and performance prediction.

In this paper, the compliance effect of the flexible membrane is explored. By altering the pore size, pressure distribution, and flow pattern, it significantly influences the mass transfer behavior and separation performance in the microfiltration process. This effect triggers a competitive mechanism between pore expansion and particle deposition of the flexible membrane. This mechanism is manifested in the actual filtration process where pore expansion and particle deposition occur simultaneously, forming a complex dynamic equilibrium relationship, which in turn determines the time-evolution characteristics of the filtration performance. The total equivalent aperture variation rate can be described by the following differential equation,

(71) $$\frac{dr_{eff}}{dt}=\frac{dr_{expansion}}{dt}-\frac{dr_{fouling}}{dt}$$

Among them, the aperture expansion rate term is based on the aperture expansion kinetics and established as follows,

(72) $$\frac{dr_{expansion}}{dt}=\frac{C_{f}\cdot r_{0}}{\tau }\cdot \frac{d\left(\Delta P\right)}{dt}$$

In the equation, τ represents the viscoelastic time constant of the material, and ΔP indicates the rate of pressure change. Under the initial condition $r_{eff}(t=0)=r_{0}$ and the steady-state condition $\frac{dr_{expansion}}{dt}=0$ when $\frac{d\left(\Delta P\right)}{dt}=0$, it can be seen that the rate of pore expansion is directly proportional to the rate of pressure change. The viscoelastic time constantly determines the response speed of the system, while the compliance parameter $C_{f}$ affects the extent of expansion.

The particle deposition rate is described by the following deposition kinetics equation:

(73) $$\;\;\frac{dr_{foulinq}}{dt}=k_{dep}\cdot C_{bulk}\cdot J\cdot \frac{1}{r_{eff}}$$

Here, $k_{dep}$ represents the deposition rate constant, $C_{bulk}$ is the particle concentration in the bulk solution, and J is the permeation flux. The term $\;\frac{1}{r_{eff}}$ indicates that the smaller the pore size, the more prone it is to be clogging. When $r_{eff}\leq d_{particle}$ the pore will be completely blocked.

Therefore, the compliance effect can to some extent counteract the impact of particle contamination. In the filtration operation, there exists optimal operating pressure, and the material selection needs to strike a balance between stiffness and anti-contamination performance. The fundamental reason lies in that when the pore expansion rate is equal to the particle deposition rate, the system reaches a dynamic equilibrium state, that is,

(74) $$\;\frac{C\cdot r_{0}}{\tau }\cdot \frac{d\left(\Delta P\right)}{dt}=k_{dep}\cdot C_{bulk}\cdot J\cdot \frac{1}{r_{eff}}$$

Under constant operating conditions, the system tends to reach a steady state, and the corresponding expression for the steady-state effective pore diameter is:

(75) $${\;\;\;\;\;r}_{eff,ss}=r_{0}\sqrt{1+\frac{C\cdot \Delta P\cdot k_{dep}\cdot C_{bulk}\cdot J\cdot \tau }{r_{0}}}$$

This model provides a theoretical basis for understanding the dynamic behavior of compliant membranes in microfiltration processes, which is helpful for optimizing operational parameters and material design.

As shown in Figure 5g,h, Gervais et al. [53] and Elodie Solnier et al. [71], respectively, verified through experiments the deformation of microchannels mainly composed of elastic material PDMS under the influence of compliance (conformity), as well as the regulation effect of this deformation on the vortex field within the microchannels.

Figure 5g presents the results of three-dimensional imaging of the deformed channel based on confocal microscopy (Zeiss LSM 510) combined with FITC-BSA fluorescence labeling. In the experiment, an accurate confocal image positioning method was adopted, with a set of 20-micron-wide triangular marks placed every 500 microns on the channel wall to compare the deformation of microfluidic channels with the same geometric structure but different material properties (elastic/non-elastic) in the narrow regions (250 microns or 500 microns). The experimental parameters were set as follows: channel width 250 mm, thickness 26 mm, length 1 cm, and Young’s modulus E = 2.2 megapascals. Among them, the upper channel was subjected to a flow rate of 300 milliliters per minute (Reynolds number Re = 13). In the subsequent numerical simulation, the channel geometry was set to 25 mm × 500 mm × 1 cm (Re = 15), with Young’s modulus E = 1 MPa. Under a pressure drop of 1 bar, the velocity field (indicated by color) and pressure field (indicated by grayscale) were analyzed through three-dimensional visualization. The simulation results show that the gradual reduction in the cross-sectional area along the channel axis leads to changes in the flow distribution and an increase in flow velocity.

Figure 5h further reveals the influence of deformation caused by compliance on the formation of vortices in microchannels through another set of experimental designs. This experiment utilized a straight channel (width Wc = 60 mm, height H = 70 mm, length L = 4 mm), and 8 reservoirs (width WR = 560 mm, length LR = 1210 mm) were set at intervals of 1 mm downstream. A filter was placed at the inlet to prevent the clogging of the channel by particle aggregates. The vortices were visualized using 1 mm fluorescent particles, with the flow rate set at 400 mL/min (Reynolds number RC based on the channel width = 156). By comparing the results of elastic materials, non-elastic materials, and numerical simulations, the relationship between the vortex length (L-vortex) and the position of the reservoirs can be clearly observed.

### 2.3. Research Advances in the Compliance Behavior of Elastic Materials

**Figure 6:**
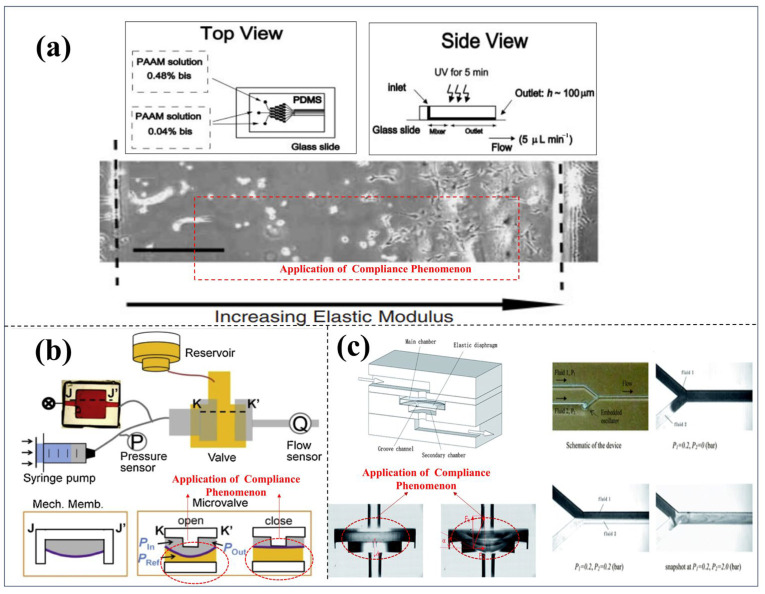
Research on the application of the compliance of elastic materials. (**a**) Microbial cell growth experiments under different surface compliance gradients. Reproduced with permission from ref. [54] Copyright (2004) WILEY-VCH. (**b**) Design of Microfluidic Single-Valve Oscillator under Compliance Effect. Reproduced with permission from ref. [55] Copyright (2019). Elsevier B.V. (**c**) Microscale compliance-driven fluid–structure interaction converts stable laminar flow into oscillatory flow. Reproduced with permission from ref. [56] Copyright (2012). Royal Society of Chemistry.

In the previously mentioned content, the compliance phenomenon of elastic materials may have adverse effects in various operation processes of LOC (lab-on-a-chip systems). However, through in-depth research, this phenomenon has also been found to have many potential positive engineering application values. As shown in Figure 6a, Zaari et al. reported a method of using photo-polymerization to regulate the compliance of the matrix in a microfluidic gradient generator, which precisely controls the flexibility of the matrix at the microscale to regulate cell behavior [54]. In Figure 6b, Xia et al. [56] proposed a microfluidic oscillator based on fluid elasticity, which can transform the originally stable laminar flow into periodic oscillating flow. This oscillator introduces an elastic membrane to enhance the nonlinear response of the fluid, thereby achieving flow pattern regulation [56]. Figure 6c shows a new passive flow regulator based on the compliance of elastic materials, whose structure is composed of five stacked functional layers. To deeply understand its flow regulation characteristics, researchers conducted systematic studies on various prototype devices with different structural dimensions [50].

In recent years, an increasing number of researchers have begun to actively explore how to utilize the compliance of elastic materials to achieve new functions and enhance device performance. Significant progress has been made in this field. Among them, using the compliance of channels to achieve automatic flow regulation is an important application direction. The basic working principle of achieving automatic flow regulation by utilizing its compliance is that the channel expands when the flow rate is high, reducing the flow resistance; when the flow rate is low, the channel contracts, increasing the flow resistance, thereby achieving automatic flow stabilization. The mathematical model it follows is as follows:

(76) $$Q=K\cdot \Delta P^{n}\cdot f\left(E,\nu ,geometry\right)$$

Among them, n and f are determined by the material and geometric parameters.

The key points of material compliance-based design also lie in:

1. The selection of material elastic modulus;
2. The optimization of channel geometry;
3. The adjustment of response characteristics.

Chappel [93] provided a comprehensive review of the development of passive flow regulators over the past three decades, systematically covering their working principles, manufacturing methods, performance characteristics, and potential applications. The review emphasized the use of elastic material compliance, which does not require external control or energy consumption, allowing these devices to maintain a constant flow rate under pressure changes. This is of great significance for applications such as drug delivery, flow chemistry, point-of-care testing, and microanalysis. Xinjie Zhang and Zhenyu Zhang [94] proposed a passive flow regulation device integrating two functional components, a flow regulation valve and a flow check valve, achieving enhanced flow control. This passive microvalve offers attractive advantages in cost-effectiveness and miniaturization for microfluidic applications, providing an important technical foundation for the commercialization of microfluidic systems.

The compliance effect in microfluidics has a significant impact on traditional particle separation methods such as deterministic lateral displacement (DLD) and inertial microfluidics, presenting both new opportunities and challenges. Its advantages mainly lie in the following three aspects:

1. Enhanced adaptive performance: The compliance effect enables microfluidic systems to automatically adjust separation parameters according to fluid conditions, thus achieving switching between different separation modes with a single device. Flexible structures have a stronger adaptability to changes in operating conditions.
2. Improved functional performance: By controlling the degree of channel deformation, real-time adjustment of separation parameters can be achieved; at the same time, soft interfaces can effectively reduce shear stress on biological samples, enhance biocompatibility, and be more easily integrated with other microfluidic functional modules.
3. Cost-effectiveness advantage: Flexible materials such as PDMS have lower processing costs, and flexible structures are less prone to damage from particle impact, making them more suitable for large-scale manufacturing and reuse.

However, through objective analysis, the compliance effect also brings some limitations, such as:

1. Nonlinear response characteristics: The relationship between channel deformation and fluid conditions is complex, making it difficult to precisely model and predict;
2. Material viscoelasticity influence: Materials like PDMS exhibit response lag, affecting the dynamic response speed of the system;
3. High pressure control requirements: The operating pressure range must be strictly controlled, as excessive pressure may lead to excessive deformation and thereby affect separation performance;
4. Difficulty in design optimization: Due to the need to consider both fluid mechanics and solid mechanics behaviors simultaneously, the parameter space dimension is high, posing significant challenges for optimization design;
5. Lack of unified standards: Currently, there is a lack of unified design guidelines and evaluation systems, which limits the standardized development of its applications in areas such as biological cell separation and particle material manufacturing.

In summary, this paper mainly discusses the compliance property of elastic materials from the perspective of passive separation technology in microfluidic chip systems (LOC) and introduces various applications with compliance characteristics represented by PDMS, combining the relevant mechanisms of fluid mechanics and elasticity mechanics. However, essentially, the compliance phenomenon can be regarded as the fluid–solid coupling effect occurring between the fluid in microchannels and elastic materials in microfluidic systems. This effect is more applicable to the explanation of nonlinear fluid elasticity mechanics theory at the mechanism level.

## 3. Compliance Influenced by Microchannel Surface Wettability

**Figure 7:**
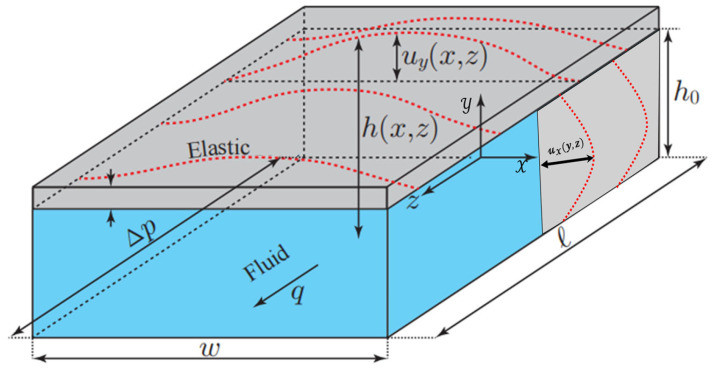
Schematic of compliance-induced side wall deformation in microchannels.

The application of microfluidic technology in physics, chemistry and biomedical sciences is becoming increasingly widespread, and it has attracted much attention due to its ability to provide higher analytical accuracy, sensitivity and efficiency in miniaturized systems. Sun et al. [95] have pointed out that at the microscale, the interaction between fluid and structure exhibits different characteristics from those at the macroscale, especially when the microchannels are made of soft materials. In microfluidic devices, laminar flow in channels made of soft materials can cause deformation of the channel geometry, which in turn affects the flow-pressure drop relationship. As shown in Figure 7, Christov et al. [96] this phenomenon is particularly significant in microchannels made of soft materials such as PDMS (polydimethylsiloxane), where pressure-driven flow can lead to obvious deformation of the channel walls. However, with the continuous development and application of surface wetting engineering, the importance of microchannel technology in microfluidic devices, biomedical engineering and energy systems has been increasingly highlighted. Surface wettability, as a key factor influencing the fluid behavior in microchannels, has a significant impact on the compliance of microchannels.

From the perspective of interface mechanism, surface wettability affects the compliance of microchannels through various physical and chemical mechanisms, mainly reflected in fluid–solid interface interaction, pressure distribution, and deformation response. Surface wettability directly determines the size of the contact angle at the liquid-solid interface. Hydrophilic surfaces (contact angle < 90°) facilitate the spreading of liquids, while hydrophobic surfaces (contact angle > 90°) inhibit liquid spreading [97]. This difference leads to different interfacial tension distributions, thereby influencing the stress state of the microchannel walls. Due to the higher adhesion energy of hydrophobic surfaces, as the number of hydrophobic surfaces increases, the flow rate shows a downward trend, and this adhesion energy difference directly affects the distribution of fluid forces on the channel walls [98].

Then, in terms of the mechanism of flow resistance, superhydrophobic surfaces can produce a significant slip effect, reducing flow resistance [99]. The increase in slip length reduces the wall shear stress, thereby reducing the mechanical load on the microchannel walls. Under hydrophobic conditions, the product of the friction coefficient and the Reynolds number increases with the increase in the Reynolds number [100], indicating that wettability alters the interaction strength between the fluid and the wall.

In addition, capillary effect is another key mechanism by which wettability influences the compliance of microchannels. Ayantika Sett et al. [101] found through the research on the surface of PDMS microchannels treated by oxygen plasma that the change in surface wettability significantly affects the magnitude and direction of capillary driving force. Hydrophilic surfaces can generate negative capillary pressure (manifested as inward contraction), while hydrophobic surfaces generate positive capillary pressure (manifested as outward expansion), forming a sharp contrast. This pressure difference directly acts on the microchannel wall and becomes one of the important factors in regulating compliance.

Finally, the research on fluid slip effect further reveals the regulatory mechanism of wettability on flow resistance. Wang et al. [99] observed significant slip phenomena in superhydrophobic microchannels, and the increase in slip length effectively reduces the wall shear stress. This reduction in shear stress not only improves flow efficiency but also reduces the mechanical load on the microchannel wall, thereby having a significant impact on its deformation characteristics.

In practical applications, the regulation of surface wettability provides a new approach for optimizing the compliance of microchannels. Zhou et al. [102] found in their study on microchannel boiling heat transfer that surfaces with different wettability significantly affect the bubble nucleation and growth process, and this influence is achieved by altering the local pressure distribution, which in turn affects the deformation behavior of the channel. Similarly, Anoop and Sen [103] confirmed in their research on deformable wall microchannels that there is a strong coupling relationship between wall deformation and surface characteristics. The study of wettability gradient surfaces has provided new ideas for passive fluid control. Funayama et al’.s review [104] pointed out that by designing specific wettability gradients, precise control over fluid motion can be achieved. This control is not only reflected in the flow direction and speed, but more importantly, it regulates the deformation response of the microchannel by changing the pressure distribution pattern.

In terms of theoretical modeling, Christov et al. [96] established a model for the flow rate-pressure drop relationship of deformable microchannels, providing a theoretical basis for understanding the coupling mechanism between wettability and compliance. These models reveal complex interactions among surface properties, fluid properties, and geometric deformation.

**Figure 8:**
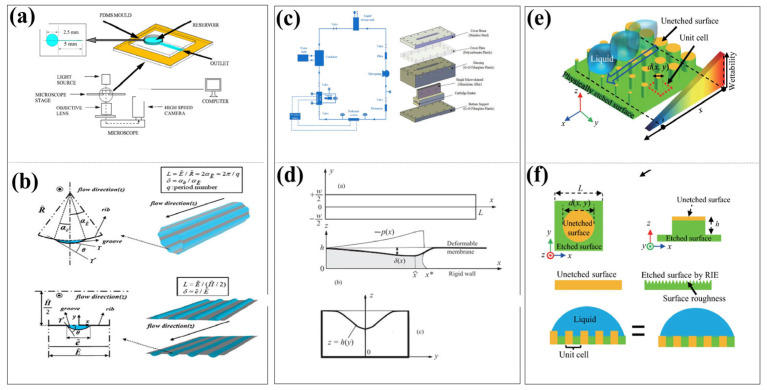
The influence of surface wettability on compliance. (**a**) Capillary Driven Flow in Wettability Altered Microchannel. Reproduced with permission from ref. [101] Copyright (2017) American Institute of Chemical Engineers. (**b**) Schematic diagram of the influence of interface deformation in superhydrophobic microtubes on flow. Reproduced with permission from ref. [99] Copyright (2014). Springer Nature. (**c**) Flow Boiling Heat Transfer in Microchannels with Different Wetting Properties: Experimental Design. Reproduced with permission from ref. [102] Copyright (2021). MDPI AG. (**d**) Numerical and Mechanistic Analysis of Enhanced Capillary Flow in Deformable Rectangular Polymer Microchannels. Reproduced with permission from ref. [103] Copyright (2015). American Physical Society. (**e**,**f**) Experimental Design and Mechanism Diagram of Tunable Wettability Gradient Based on Physical Surface Modification for Passive Fluid Control. Reproduced with permission from ref. [104] Copyright (2023). Springer Nature.

Therefore, as shown in Figure 8, the wettability of the microchannel surface affects the compliance of the microchannel through multiple mechanisms such as interfacial interaction, capillary effect, slip phenomenon, and pressure distribution regulation. This influence is not only reflected in static geometric deformation but more importantly in the fluid–structure coupling response during dynamic flow processes. Therefore, future research should focus on the in-depth understanding of multi-scale coupling mechanisms and the engineering application of intelligent surface design.

## 4. Conclusions

Microfluidic systems can rapidly identify pathogenic microorganisms or harmful particles in water systems, thereby providing urgently needed innovative methods for implementing preventive measures and enhancing the productivity of aquaculture. By regulating the channel structure at the micro-nano scale, microfluidic technology can precisely control fluid flow patterns, offering new insights and effective solutions for microbiological research as well as the separation and analysis of particulate matter. This article provides an overview of the application of microfluidic chip technology in marine microbiological analysis, with a focus on the “compliance” phenomenon in PDMS-based microfluidic systems. It systematically reviews the potential mechanisms, latest progress, and impacts of compliance behavior in mechanically elastic materials such as PDMS. Through mechanistic formula analysis, the influence and role of “compliance” in key processes of microfluidic technology applications are explored, mainly in the separation, filtration, and screening of microorganisms and particles. Finally, the relationship between surface wettability engineering and the compliance phenomenon is also discussed.

This paper reviews the phenomenon of “compliance” in microfluidic systems and its influences. Based on the fluid dynamics mechanism analysis, the following conclusions are drawn:

1. At the microscale, the interaction between fluid and structure exhibits distinct characteristics from those at the macroscale, especially when the microchannels are made of flexible materials.
2. In microfluidic devices, the flow of laminar flow in flexible material channels can cause deformation of the channel geometry, and this deformation in turn affects the relationship between flow rate and pressure drop. This phenomenon is particularly evident in microchannels made of flexible materials such as PDMS (polydimethylsiloxane), where pressure-driven flow can lead to significant deformation of the channel walls.
3. The fluid mechanics effects at the microscale or mesoscale are unique: the small characteristic length scale and high deformation rate can trigger significant stretching flow effects at the constricted throat, and even dilute polymer solutions with millisecond time constants can exhibit strong viscoelastic behavior. By introducing the definition of the elastic number El = Wi/Re, it can be known that the length scale of the geometric structure is a key factor in triggering strong viscoelastic effects.
4. In practical applications, the compliance effect brings both challenges and opportunities, involving the design optimization of various microfluidic devices such as micro-mixers, micro-heat exchangers, logic microfluidic circuits, and particle manipulation.
5. Numerical simulation helps deepen theoretical understanding, reduce prototype testing, and accelerate the development process. However, the complexity of multi-physics field coupling must be fully considered. For fluid–solid coupling problems in microfluidic channels, advanced numerical methods should be adopted to accurately capture the interactions among various physical fields. These methods need to handle the flow behavior in continuously deforming geometries and the resulting deformations and displacements. Meanwhile, modeling particles with elastic deformation (such as bacteria) as spheres is a simplification that ignores the natural differences in size and shape of bacteria. This assumption’s limitations should be acknowledged [105]. Due to the wide variety of bacteria and microorganisms in nature, their diverse shapes, and the possible dynamic interactions among them in in situ detection, as pointed out by a renowned research team [106]. Additionally, in viscoelastic fluids, the collective behavior of bacteria and the coupling effects between turbulence and individual bacteria are also key factors to be considered in future application research [107,108].
6. Further exploration by authors and experts in related fields is still needed in obtaining analytical solutions for compliance phenomena caused by fluid–structure interaction and in-depth research on the nonlinear fluid elastic mechanics mechanism.

## Data Availability

The original contributions presented in the study are included in the article, further inquiries can be directed to the corresponding author.
